# Diaphragmatic Surgery and Related Complications In Primary Cytoreduction for Advanced Ovarian, Tubal, and Peritoneal Carcinoma

**DOI:** 10.1186/s12885-017-3311-8

**Published:** 2017-05-05

**Authors:** Shuang Ye, Tiancong He, Shanhui Liang, Xiaojun Chen, Xiaohua Wu, Huijuan Yang, Libing Xiang

**Affiliations:** 1 Department of Gynecologic Oncology, Fudan University Shanghai Cancer Center, Shanghai, 200032 China; 2 Department of Oncology, Shanghai Medical College, Fudan University, Shanghai, 200032 China

**Keywords:** Ovarian carcinoma, Diaphragm, Surgery, Complications

## Abstract

**Background:**

To evaluate the procedures and complications of diaphragm peritonectomy (DP) and diaphragm full-thickness resection (DFTR) during primary cytoreduction for advanced stage epithelial ovarian cancer.

**Methods:**

All the patients with epithelial ovarian carcinoma who underwent diaphragm procedures at our institution between January 2009 and August 2015 were identified. Clinicopathological data were retrospectively collected from the patients’ medical records. Postoperative morbidities were assessed according to the Memorial Sloan-Kettering Cancer Center (MSKCC) grading system.

**Results:**

A total of 150 patients were included in the study. The majority of the patients had ovarian cancer (96%), stage IIIC disease (76%) and serous histology (89.3%). DP and DFTR were performed in 124 (82.7%) and 26 (17.3%) patients, respectively. A total of 142 upper abdominal procedures in addition to the diaphragmatic surgery were performed in 77 (51.3%) patients. No macroscopic residual disease was observed in 35.3% of the patients, while 84% of the total patient cohort had residual disease ≤1 cm. The overall incidence of at least one major morbidity (MSKCC grades 3–5) was 18.0%, whereas pleural effusions (33.3%), pneumonia (15.3%) and pneumothorax (7.3%) were the most commonly reported morbidities. The rate of postoperative pleural drainage was 14.6% in total, while half the patients in the DFTR group received drainage intraoperatively (11.5%) and postoperatively (38.5%). The incidence of postoperative pleural effusion was associated with stage IV disease (hazard ratio [HR], 17.2; 95% confidence interval [CI]: 4.5–66.7; *P* < 0.001), DFTR (HR, 4.9; 95% CI: 1.2–19.9; *P* = 0.028) and a long surgery time (HR, 15.4; 95% CI: 4.3–55.5; *P* < 0.001).

**Conclusions:**

Execution of DP and DFTR as part of an extensive upper abdominal procedure resulted in an acceptable morbidity rate. Pleural effusion, pneumonia and pneumothorax were the most common pulmonary morbidities. The pleural drainage rate was not high enough to justify prophylactic chest tube placement for all the patients. However, patients who underwent DFTR merited special consideration for intraoperative prophylactic drainage.

## Background

Epithelial ovarian carcinoma is the most lethal gynecologic malignancy [1]. A recent publication from China reported that approximately 52,100 new cases of ovarian cancer were diagnosed in 2015 and that 22,500 women will die from this disease [2]. Most patients present with advanced stage disease, and optimal cytoreduction has been shown to be the cornerstone of effective treatment [3, 4]. Patients with advanced ovarian cancer often develop metastatic disease in the upper abdominal region, and extensive upper abdominal procedures are advocated as part of the surgical armamentarium [5]. Of note, it is estimated that nearly 40% of patients with ovarian cancer have widespread disease in the diaphragm [6, 7].

In the past decade, several important studies (primarily from the United States and European countries) focusing on surgical diaphragm procedures have been published [8–18]. In some of these studies, ablative procedures (e.g., Cavitron ultrasound aspiration or argon beam coagulation) were also included [13–16].

In China, only a few gynecologic oncologists are willing to perform extensive upper abdominal surgery due to either a lack of the relevant surgical skills or the intense patient-physician relationship [19]. Since January 2009, upper abdominal procedures have been incorporated into the primary cytoreduction at the Fudan University Shanghai Cancer Center. Our previous publication reported that the overall number of major complications accompanying radical upper abdominal surgery were acceptable [19]. The current study was conducted to specifically assess diaphragmatic surgery in primary cytoreduction for patients with advanced ovarian, tubal and peritoneal cancer. The perioperative complications were also evaluated in relation to diaphragm surgery.

## Methods

### Study patients

This study was approved by the institutional review board (SCCIRB-090371-2). After we searched the electronic medical record database, we identified all the patients with epithelial ovarian cancer who underwent either diaphragm peritonectomy (DP; stripping) or diaphragm full-thickness resection (DFTR) in primary cytoreduction between January 2009 and August 2015. A comprehensive retrospective review of available medical documentation was performed by two gynecologic oncologists. All the included patients provided their written informed consent.

### Diaphragmatic surgery

**Fig. 1:**
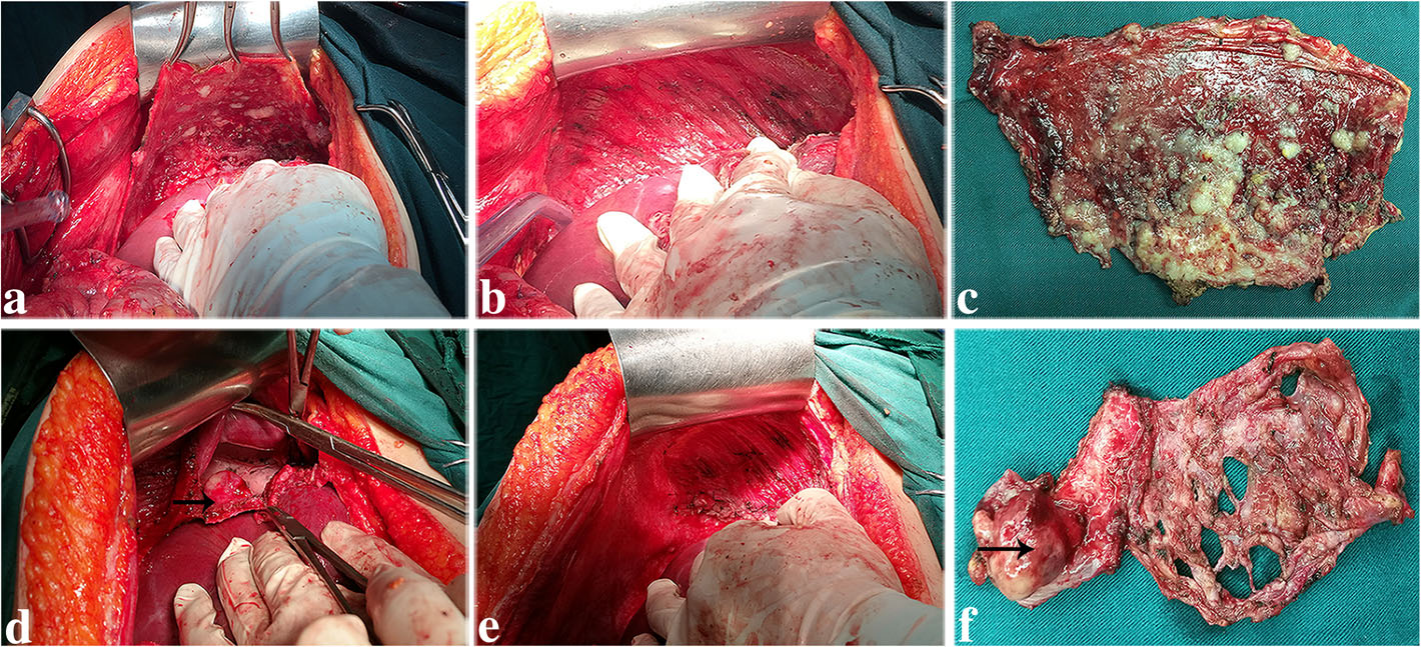
Diaphragm peritonectomy (**a**-**c**) and full-thickness resection (**d**-**f**). **a**-**b** represents the status before and after peritonectomy. **c** shows the sample. **d** presents that the tumor infiltrated into the diaphragm muscle and the nodule in the pleural cavity was pointed out by arrow. **e** illustrates the diaphragm after resection and repair. **f** shows the diaphragm sample

The incision was extended to the xiphoid process for adequate exposure and space. A fixed retraction device was employed to elevate the costal margin. Falciform ligament dissection was an essential procedure for providing extensive exposure for diaphragm exploration. Dissection was extended to the coronary and triangular ligaments for complete liver mobilization. After the lesions were evaluated, either DP or DFTR was performed on the basis of muscle infiltration. DP (Fig. 1 a-c) is defined as dissection of the overlying peritoneum, while DFTR (Fig. 1 d-f) refers to resection of the diaphragm muscle inclusive of the overlying peritoneum and pleura. The extent of the procedure was determined by the distribution of the tumor lesions. Monopolar cautery was applied to perform the diaphragmatic procedure. Several key points of the specific surgical technique are mentioned here. Before diaphragm resection, the diaphragm is gripped with several clamps to separate the diaphragm muscle from the overlying lung tissue. After resection, exploration of the pleural cavity with fingers is routinely performed in order to confirm the extent of resection. With regard to DP, the central tendon merits special attention to avoid incidental rupture of this weak structure as much as possible. Identification and protection of the right hepatic vein is also significant because this vein drains into the anterior surface of inferior vena cava at the level where coronary ligaments reflect off the liver capsule. Therefore, special attention should be taken to avoid injury to these major vessels during the dissection. It is also essential to be cautious and avoid tearing the right hepatic vein when pushing downward on the liver. The diaphragm defect was closed with a large-caliber un-absorbable suture. The anesthesiologist was asked to give the patient maximal inspiration, and the final diaphragmatic suture was tied down. After the diaphragmatic surgery, a bubble test was performed to identity any possible defect in the diaphragm [20]. The application of either mesh reconstruction or a prophylactic chest tube was at the discretion of the operating surgeons. Trans-diaphragmatic thoracic exploration (TDDE) was performed in some patients [21]. Specific indications for TDDE were presented in the previous publication. The definition of the extended procedures in addition to the diaphragmatic surgery was in line with our previous publication [19].

### Perioperative morbidities

Perioperative morbidities and mortality were defined as any adverse events within 30 days of surgery that were related to treatment. All perioperative complications in the current series were graded according to the Memorial Sloan-Kettering Cancer Center (MSKCC) surgical secondary events grading system [22, 23]. Grade 3–5 complications are those that lead to invasive reoperation, unplanned intensive care unit (ICU) admission, chronic disability or death [23].

Patients presenting with no physical signs or symptoms of pulmonary complications were exempted from subsequent routine chest radiographs. The definition of ipsilateral effusions was effusions on the same side as the diaphragm operation. In patients with pleural effusions preoperatively, an increase in the size of the effusion (comparison of chest X-ray before and after operation if indicated) was included as a positive finding. The laterality and size (small, moderate or large as determined by imaging modality) of the effusions were recorded.

### Data collection and statistical analyses

In our center, preoperative work up for patients highly suspicious for ovarian cancer involved serum tumor marker, a comprehensive radiologic imaging (thorax/abdomen/pelvis), and gastroscopy and colonoscopy if necessary. Patient-, disease- and surgery-related information was extracted from the patients’ medical records. The data collection included age at diagnosis, primary site of disease, body mass index (BMI, calculated as weight (kg)/[height (m)]^2^), histological subtype, International Federation of Gynecology and Obstetrics (FIGO) stage [24], the presence of ascites and pleural effusion at the time of disease diagnosis, and administration of neoadjuvant chemotherapy. Preoperative laboratory values, including serum protein (i.e., total protein and albumin), and serum cancer antigen 125 (CA-125), were also recorded. The surgery-related parameters were listed as follows: operation radicality, distribution of diaphragm implants, diaphragm surgery type (DP or DFTR), perforation into the pleural cavity, mesh application during diaphragm repair, prophylactic chest tube placement, residual disease, operation time, estimated blood loss (EBL), intra-operative transfusion, ICU stay, postoperative complications, and time interval from surgery to chemotherapy. Preoperative plural or peritoneal effusions were drained only if the patients had any related symptoms. In concordance with the Gynecologic Cancer InterGroup (GCIG) consensus, optimal cytoreduction refers to no macroscopic residual disease [25].

Parametric Student’s *t*-tests were employed in evaluating continuous variables, while chi-square tests were used for the categorical variables. The associations between different variables were evaluated using univariate and multivariate logistic regression analyses, and the hazard ratio (HR) with 95% confidence interval (CI) was calculated. All of the *P* values reported were two-sided, and a value of *P* < 0.05 was considered statistically significant. Statistical Package for Social Science (SPSS) (Version 17.0, SPSS, Inc., Chicago, IL, USA) and GraphPad Prism (Version 5.0, GraphPad Software, Inc., La Jolla, CA, USA) were used for all the analyses.

## Results

**Fig. 2:**
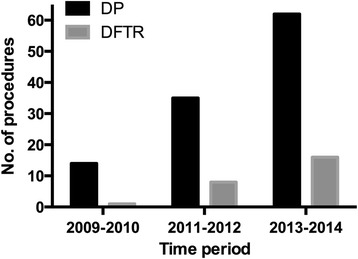
Trends in utilizing diaphragmatic procedures for advanced ovarian, tube, and peritoneal carcinoma at the Fudan University Shanghai Cancer Center. Abbreviations: DP = diaphragm peritonectomy; DFTR = diaphragm full-thickness resection; DFTR% = percentage of DFTR in the total population

A total of 150 patients underwent diaphragmatic surgery. Figure 2 highlights the increasing application of diaphragmatic surgery at our institution over the past 6 years. The patient characteristics of the entire cohort are shown in Table 1. The median age was 55 years (range, 25–77 years). The majority of the patients had ovarian cancer (96%), FIGO stage IIIC tumor (76%) and serous histology (89.3%). Neoadjuvant chemotherapy was administered in 14 (9.3%) patients. Ascites was present in 94% of the patients, and the median volume was 2000 mL (range, 20–7300 mL). Before surgery, it was noted that 47 (31.3%) patients had pleural effusions, which were distributed as right-sided (9, 6.0%), left-sided (9, 6.0%), and bilateral (29, 19.3%). Among these patients, seven symptomatic patients (4.7%) underwent preoperative pleural drainage.

**Table 1:** Patient baseline characteristics of the entire cohort

Variables	
Median age (range), years	55 (25–77)
Median body mass index (range), kg/m^2^	22.7 (15.0–32.9)
Site of disease	
Ovary (%)	144 (96%)
Fallopian tube/peritoneum (%)	6 (4%)
Neo-adjuvant chemotherapy (%)	14 (9.3%)
Preoperative laboratory values	
Median CA-125 (range), U/mL	1166 (57–5502)
Median total protein (range), g/dL	7.1 (4.1–9.6)
Median albumin (range), g/dL	3.9 (2.4–8.2)
Tumor stage	
IIIC (%)	114 (76%)
IV (%)	36 (24%)
Histology	
Serous (%)	134 (89.3%)
Non-serous (%)	16 (10.7%)
Presence of pleural effusion before surgery (%)	47 (31.3%)
Right	9 (6.0%)
Left	9 (6.0%)
Bilateral	29 (19.3%)
Preoperative pleural drainage (%)	7 (4.7%)
Presence of ascites at surgery (%)	141 (94%)
Median ascites volume (range), mL	2000 (20–7300)
Type of diaphragm surgery	
Diaphragm peritonectomy (%)	124 (82.7%)
Diaphragm full-thickness resection (%)	26 (17.3%)

*Abbreviations*: *CA-125* cancer antigen 125

DP and DFTR were performed in 124 (82.7%) and 26 (17.3%) patients, respectively. Table 2 lists the specific surgical procedures and outcomes based on the diaphragmatic surgery stratification. The diaphragm lesions were predominantly right-sided (63.3%) followed by bilateral (36.0%). We did notice one case with only left hemidiaphragm involvement. The diaphragm was opened during 65 (43.4%) procedures, while TDDE was performed in 34 (22.7%) patients. Of these 34 patients, suspicious pleural lesions were noted in 21 (61.8%) patients; therefore, a biopsy was collected. Mesh was utilized in four patients (2.7%) when closing the diaphragm opening after DFTR. Intraoperative chest tube placement was conducted in eight (5.3%) patients: five (4.0%) in the DP group and three (11.5%) in the DFTR group (*P* = 0.285). Extended procedures in addition to the diaphragmatic surgery were performed in 77 (51.3%) patients, resulting in a total of 142 procedures. The specific details of these procedures are shown in Table 2. The debulking results were 53 (35.3%) patients with no gross residual disease, 73 (48.7%) with gross residual disease ≤1 cm, and 24 (16.0%) with gross residual disease >1 cm. The two patient groups (DP vs. DFTR) had no difference with regard to the extended procedures and cytoreduction outcomes.

**Table 2:** Surgical procedures and outcomes based on type of diaphragm surgery

	Cohort (*n* = 150)	DP (*n* = 124)	DFTR (*n* = 26)	*P*
Laterality of diaphragm lesions (%)				
Right	95 (63.3%)	78 (62.9%)	17 (65.4%)	0.811
Left	1 (0.7%)	1 (0.8%)	0	
Bilateral	54 (36.0%)	45 (36.3%)	9 (34.6%)	
Perforation into pleural cavity (%)	65 (43.3%)	39 (31.5%)	26 (100%)	**<0.001**
TDDE (%)	34 (22.7%)	21 (16.9%)	13 (50.0%)	**<0.001**
Pleural nodule biopsy performed (%)	21 (14.0%)	15 (12.1%)	6 (23.1%)	0.248
Prophylactic chest tube placement (%)	8 (5.3%)	5 (4.0%)	3 (11.5%)	0.285
Mesh utilization in diaphragm repair (%)	4 (2.7%)	0	4 (15.4%)	**<0.001**
Extended procedures performed (%)				
Small bowel resection	5 (3.3%)	5 (4.0%)	0	0.660
Large bowel resection	48 (32.0%)	37 (29.8%)	11 (42.3%)	0.215
Splenectomy	18 (12.0%)	14 (11.3%)	4 (15.4%)	0.801
Partial pancreatectomy	5 (3.3%)	4 (3.2%)	1 (3.8%)	1.000
Partial hepatic resection	4 (2.7%)	4 (3.2%)	0	0.796
Resection of porta hepatis tumor	4 (2.7%)	3 (2.4%)	1 (3.8%)	1.000
Cholecystectomy	1 (0.7%)	1 (0.8%)	0	1.000
Stomia	15 (10.0%)	12 (9.7%)	3 (11.5%)	1.000
Resection of the tumor on the liver surface	23 (15.3%)	20 (16.1%)	3 (11.5%)	0.771
Resection of the tumor on the stomach surface	13 (8.7%)	10 8.1(%)	3 (11.5%)	0.850
Resection of the tumor in the gallbladder fossa	6 (4.0%)	6 (4.8%)	0	0.552
Residual disease (%)				
Complete cytoreduction	53 (35.3%)	42 (33.9%)	11 (42.3%)	0.649
Residual disease ≤1 cm	73 (48.7%)	61 (49.2%)	12 (46.2%)	
Median operation time (range), minutes	180 (60–330)	180 (60–330)	195 (90–270)	0.576
Median EBL (range), mL	900 (100–5300)	900 (100–5300)	1000 (200–2200)	0.802
Intraoperative blood transfusion (%)	132 (88%)	109 (87.9%)	23 (88.5%)	0.937
Median transfusion amount (range), units	4.0 (1.0–15.0)	4.5 (1–15)	4 (2–13)	0.286
Planned intensive care unit stay (%)	46 (30.7%)	39 (30.6%)	8 (30.8%)	0.649
Median time interval from surgery to chemotherapy (range), days	14 (6–40)	14 (6–40)	14 (6–34)	0.272

Note: Values in bold are statistically significant.

*Abbreviations*: *DP* diaphragm peritonectomy, *DFTR* diaphragm full-thickness resection, *TDDE* trans-diaphragmatic thoracic exploration, *EBL* estimated blood loss

The median operation time was 180 min (range, 60–330 min), while the median blood loss was 900 mL (range, 100–5300 mL). Intraoperatively, 88% of the patients received a transfusion, and the median volume transfused was 4.0 units (range, 1–15 units). In all, 46 (30.7%) patients had a planned transient postoperative ICU stay. For the entire cohort, the median time from surgery to chemotherapy was 14 days (range, 6–40 days). No significant difference was observed between the DP and DFTR groups with regard to these characteristics (*P* = 0.272).

Table 3 is a comprehensive review of the postoperative complications. Pleural effusions and pneumothorax occurred in 50 (33.3%) and 11 (7.3%) patients, respectively. Ten of the 11 patients had concurrent effusions, while only one patient developed an exclusive pneumothorax. In other words, a total of 51 patients developed postoperative pleural effusions and/or pneumothorax. Pneumonia was the main concurrent finding based on the postoperative imaging. Neither diaphragmatic hernia nor hydrothorax was observed in either group. Regarding the MSKCC grading system, there were 82 mild (Grade 1–2) and 27 severe (Grade 3–5) adverse events in the entire cohort. The specific details of the severe complications are listed as follows: symptomatic pleural effusion requiring drainage (21, 14.0%), symptomatic pneumothorax requiring a thoracostomy tube (1, 0.6%), right hepatic vein rupture requiring intra-operative repair and transfusion (1, 0.6%), bleeding requiring return to the operating room (1, 0.6%), pancreatic leak requiring drainage (1, 0.6%), intestinal perforation requiring return to the operating room (1, 0.6%), and wound dehiscence resulting in delayed repair (1, 0.6%). There was no mortality (MSKCC grade 5) within 30 days of surgery. Patients in the DFTR group were more likely to have postoperative pleural effusion (69.2% vs. 25.8%, *P* < 0.001) and pleural drainage (38.5% vs. 8.9%, *P* < 0.001). No significant difference was observed with the other morbidities.

**Table 3:** Perioperative surgical complications based on type of diaphragm surgery

	Cohort (*n* = 150)	DP (*n* = 124)	DFTR (*n* = 26)	*P*
All complications				
Ipsilateral pleural effusion (%)	50 (33.3%)	32 (25.8%)	18 (69.2%)	**<0.001**
Ipsilateral pneumothorax (%)	11 (7.3%)	8 (6.5%)	3 (11.5%)	0.623
Pulmonary embolism (%)	2 (1.3%)	2 (1.6%)	0	1.000
Pneumonia (%)	23 (15.3%)	20 (16.1%)	3 (11.5%)	0.771
Right hepatic vein rupture	1 (0.6%)	0	1 (3.8%)	1.000
Sub-diaphragmatic abscess	1 (0.6%)	1 (0.8%)	0	1.000
Postoperative bleeding (%)	1 (0.6%)	1 (0.8%)	0	1.000
Bowel obstruction (%)	10 (6.7%)	9 (7.3%)	1 (3.8%)	0.825
Pancreatic leak	1 (0.6%)	1 (0.8%)	0	1.000
Intestinal perforation	1 (0.6%)	1 (0.8%)	0	1.000
Heart arrhythmia	1 (0.6%)	1 (0.8%)	0	1.000
Wound infection/dehiscence	5 (3.3%)	3 (2.4%)	2 (7.7%)	0.447
Vaginal cuff infection	1 (0.6%)	1 (0.8%)	0	1.000
Urinary tract infection	1 (0.8%)	1 (0.8%)	0	1.000
MSKCC grading				
Grade 1–2 (%)	82 (54.7%)	66 (53.2%)	16 (61.5%)	**0.010**
Grade 3–5 (%)	27 (18.0%)	15 (12.1%)	12 (46.2%)	
Grade 3–5 complications^a^				
Symptomatic pleural effusion requiring drainage	21 (14.0%)	11 (8.9%)	10 (38.5%)	**<0.001**
Symptomatic pneumothorax requiring thoracostomy tube	1 (0.6%)	0	1 (3.8%)	1.000
Right hepatic vein rupture requiring intra-operative repair and transfusion	1 (0.6%)	0	1 (3.8%)	1.000
Bleeding requiring return to operating room	1 (0.6%)	1 (0.8%)	0	1.000
Pancreatic leak requiring drainage	1 (0.6%)	1 (0.8%)	0	1.000
Intestinal perforation requiring return to the operating room	1 (0.6%)	1 (0.8%)	0	1.000
Wound dehiscence requiring delayed repair	1 (0.6%)	1 (0.8%)	0	1.000

*Abbreviations*: *DP* diaphragm peritonectomy, *DFTR* diaphragm full-thickness resection, *MSKCC* Memorial Sloan Kettering Cancer Center

Note:

1. Percentages are not additive as multiple procedures might be performed on the same patient.

2. Values in bold are statistically significant.

^a^Severe complications leading to invasive radiologic intervention/re-operation/unplanned ICU admission (grade 3), chronic disability (grade 4), or death (grade 5).

We further evaluated the application of postoperative thoracentesis and thoracostomy tube placement. Pleural effusions and pneumothorax were most commonly diagnosed on postoperative day (POD) 3 (range, 1–16 days). It was worth mentioning that of the 51 patients with postoperative pleural effusions and/or pneumothorax, only 21 (41.2%) patients had pulmonary-related symptoms. A total of 22 pleural drainages were performed in the 21 patients primarily by thoracentesis (20/22, 90.9%). Pleural puncture was performed at a median of 3 days (range, 2–13 days) postoperatively. Two patients required thoracostomy tube placement for pulmonary complications, which warrants discussion. The first patient presented with diffuse lesions in the right hemidiaphragm (approximately 8 × 6 cm) that infiltrated into the diaphragm muscle. She underwent DFTR and pleural nodule biopsy without either a prophylactic chest tube or mesh reconstruction. On POD 1, the bedside chest film revealed a right-side pneumothorax with the lung compression of nearly 30%. The patient received a thoracic surgical consultation, and a thoracostomy tube was placed on the same day. On POD 2, bilateral pleural effusions were noted in the bedside ultrasonography, and thoracentesis was performed. The second patient received right-sided DP for multiple small nodules without either a pleural opening or chest tube placement. On POD 4, the patient experienced sudden onset chest distress and dyspnea. Pulse oxygen saturation was approximately 97% on 3 L/min of nasal cannula oxygenation. Computer tomography pulmonary angiography revealed a large amount of ipsilateral hydrothorax instead of pulmonary embolism. A thoracostomy tube was inserted into the sixth intercostal space.

Table 4 illustrates the possible risk factors for pleural effusion and drainage after diaphragmatic surgery. After multivariate analysis, stage IV disease (HR, 17.2; 95% CI: 4.5–66.7; *P* < 0.001), DFTR (HR, 4.9; 95% CI: 1.2–19.9; *P* = 0.028) and a long operating time (HR, 15.4; 95% CI: 4.3–55.5; *P* < 0.001) retained their statistical significance. In contrast, DFTR (HR, 5.9; 95% CI: 1.5–23.6; *P* = 0.011) and TDDE (HR, 28.3: 95% CI: 4.9–160.8; *P* < 0.001) were found to be predictive factors for pleural drainage.

**Table 4:** Univariate and multivariate analysis of factors predictive of postoperative ipsilateral plural effusion and drainage after diaphragm surgery

Univariate				
Parameters	Pleural effusions		Pleural drainage	
	*P* value		*P* value	
Age (>55 years old)	0.908		0.080	
Tumor stage (IV vs. IIIC)	**<0.001**		**0.009**	
Preoperative pleural effusion	0.215		0.831	
Ascites >2000 mL	0.558		0.878	
CA-125 > 1166 U/mL	0.729		0.482	
Albumin <3.9 g/dL	0.568		0.815	
Diaphragmatic surgery (DFTR vs. DP)	**<0.001**		**<0.001**	
Perforation into pleural cavity	0.996		0.996	
TDDE	0997		**<0.001**	
Pleural nodule biopsy	0.998		0.995	
Operating time > 180 min	**<0.001**		**0.015**	
Estimated blood loss >900 mL	0.249		0.442	
Intraoperative blood transfusion >4.0 units	**0.016**		0.719	
Multivariate				
Parameters	Pleural effusions		Pleural drainage	
	HR (95% CI)	*P* value	HR (95% CI)	*P* value
Tumor stage (IV vs. IIIC)	17.2 (4.5–66.7)	**<0.001**	4.5 (0.8–25.7)	0.086
Diaphragmatic surgery (DFTR vs. DP)	4.9 (1.2–19.9)	**0.028**	5.9 (1.5–23.6)	**0.011**
TDDE	/	/	28.3 (4.9–160.8)	**<0.001**
Operating time > 180 min	15.4 (4.3–55.5)	**<0.001**	2.2 (0.6–8.6)	0.242
Intraoperative blood transfusion >4.0 units	0.7 (0.2–2.3)	0.581	/	/

Note: Values in bold are statistically significant.

*Abbreviations*: *CA-125* cancer antigen 125, *DP* diaphragm peritonectomy, *DFTR* diaphragm full-thickness resection, *TDDE* trans-diaphragmatic thoracic exploration, *HR* hazard ratio, *CI* confidence interval.

## Discussion

In the current series, we analyzed the results of patients with stage IIIC–IV ovarian carcinoma who underwent diaphragmatic procedures for primary cytoreduction. To the best of our knowledge, the present study is one of the largest series and the first study from a Chinese academic center [7, 26].

Adequate exposure of the diaphragm is the very first and critical step in not only assessing tumor resectability but also performing the procedure. Involvement of the right hemidiaphragm was extraordinarily common in previous publications [12, 13, 27] as well as in our study. Based on our clinical observation (although without supporting data available), bulky tumors are frequently identified in the area where the diaphragmatic peritoneum is reflected to the capsule of the posterior region of the right liver lobe. Given that large-volume disease on the right hemidiaphragm is obscured by the right liver, liver mobilization and medial retraction of the liver allow exposure of the diaphragmatic lesion. Dr. Chi from MSKCC mentioned that tumor implantation on the left diaphragm could be more easily resected without fully mobilizing the liver, although in some cases, splenectomy might be necessary [20]. In our experience, adequate mobilization and extensive knowledge of the upper abdominal anatomy are fundamental to a successful diaphragm operation.

Concerning the type of diaphragmatic surgery, we did not include ablation and coagulation procedures in this study. The two techniques (DP and DFTR) described are not comparable given that surgeons do not have a choice in which technique to perform. In our cohort, more patients underwent diaphragm stripping (82.7% vs. 17.3% for DP and DFTR, respectively), which might be explained that ovarian cancer tended to be superficially spread within the peritoneal surface [7]. Patients in the two groups were similar in terms of surgical procedures and outcomes.

The most commonly encountered adverse events were new or increased pleural effusions. The rate of intraoperative chest tube placement was 5.3%, and postoperative pleural drainage accounted for 14.6% in the entire population. Thus, we do not feel that this rate is high enough to routinely place a prophylactic chest tube in all of the patients during the operation. However, when it comes to DFTR alone, half the patients underwent drainage during the operation for prophylactic purpose (11.5%) and after the operation for relieving the symptom (38.5%). Therefore, intraoperative prophylactic tube placement should be considered for the subgroup patients. Among the 292 included patients (197 DP and 75 DFTR) from a recent systematic review, the estimated pleural effusion rates after DP and DFTR were 43% and 53%, respectively, while the need for pleural punctures or chest tube placement after DP and DFTR was 4% and 9%, respectively [7]. In the MSKCC study, the rate of ipsilateral effusions was 58%, and the overall rate of either postoperative thoracentesis or chest tube placement was 15% [10]. Researchers have investigated possible predictive factors for postoperative effusions in different populations [10, 13, 16, 17, 28]. The following parameters retained significance upon multivariate analysis: liver mobilization [10, 13], entry into the pleural space during DP [28], and the size of the diaphragmatic resection [13, 16]. Given that we routinely divide the hepatic ligaments to mobilize the liver, we were unable to test this association in our own series. Based on our data, stage IV disease, DFTR and a long operating time (>180 min) correlated with postoperative pleural effusions with statistical significance. Researchers from Italy also noticed that patients who underwent DFTR were more likely to have postoperative pleural effusions compared to patients who underwent DP [29]. The duration of surgery was recognized as a common risk factor for postoperative complications [10]. It has been proposed that complications due to diaphragmatic surgery result from the transfer of ascites to the pleural cavity rather than as primary thoracic processes [10, 15]. Diaphragm defects, the presence of ascites, extended exposure of the diaphragmatic bare area after liver mobilization and postoperative release of either VEGF or inflammatory mediators were suggested was possible mechanisms for pleural effusion [10, 15].

Despite the high reported rate of pneumothorax, symptomatic pneumothorax requiring intervention was quite low in our series. One of the reasons for this finding might be that residual pneumothorax was not evacuated by a catheter prior to diaphragmatic closure. Since 2015, we have included an intentional evacuation of the pneumothorax by suctioning as part of the surgical procedure.

When interpreting the results of this study, several potential limitations must be addressed. Firstly, this study has inherent bias pertaining to its retrospective design. Secondly, survival information was not evaluated in the present series. The short follow-up period and the number of patients who underwent multiple radical procedures were two reasons that we did not attempt to assess the survival outcome. Thirdly, the multivariate model assessing the predictive factors for postoperative drainage might overfit with factors given the few number of drainage events. Last but not least, the subject data were collected from a tertiary referral center, and although the cohort contained a relatively large sample size, the results might not be generalizable to all of the patients in China. For now, the application of upper abdominal procedures (including diaphragm surgery) for patients with ovarian cancer in China remains unclear because there are few publications focused on this issue. The importance of not only surgical skills and experience but also the high-quality surgical care delivered by a multidisciplinary team has been increasingly emphasized [16]. Referrals should be considered at institutions where the necessary treatments are unavailable.

## Conclusions

The performance of DP and DFTR as part of the extensive upper abdominal operation resulted in an acceptable morbidity rate. Pleural effusion, pneumonia and pneumothorax were the most common morbidities, and the rate of pleural drainage was not high enough to justify prophylactic chest tube placement for all the patients. However, for patients who received DFTR, intraoperative prophylactic drainage should be considered.

## Abbreviations

BMI: Body mass index
CA-125: Cancer antigen 125
CI: Confidence interval
DFTR: Diaphragm full-thickness resection
DP: Diaphragm peritonectomy
EBL: Estimated blood loss
FIGO: International Federation of Gynecology and Obstetrics
HR: Hazard ratio
ICU: Intensive care unit
MSKCC: Memorial Sloan-Kettering Cancer Center
TDDE: Trans-diaphragmatic thoracic exploration
